# Prevalence, risk factors, and medical costs of *Chlamydia trachomatis* infections in Shandong Province, China: a population-based, cross-sectional study

**DOI:** 10.1186/s12879-018-3432-y

**Published:** 2018-10-26

**Authors:** Pengcheng Huai, Furong Li, Zhen Li, Lele Sun, Xi’an Fu, Qing Pan, Gongqi Yu, Zemin Chai, Tongsheng Chu, Zihao Mi, Fangfang Bao, Honglei Wang, Bingni Zhou, Chuan Wang, Yonghu Sun, Guiye Niu, Yuan Zhang, Fanghui Fu, Xiaoqiao Lang, Xiaoling Wang, Hui Zhao, Daina Liu, Hong Liu, Dianchang Liu, Jian Liu, Aiqiang Xu, Furen Zhang

**Affiliations:** 1 Shandong Provincial Hospital for Skin Disease, Shandong University, Jinan, China; 2 Shandong Provincial Institute of Dermatology and Venereology, Shandong Academy of Medical Sciences, 27397 Jingshi Road, Jinan, 250022 China; 3 Department of Epidemiology, School of Public Health, Shandong University, Jinan, China; 4 Shandong Provincial Key Laboratory for Dermatovenereology, Jinan, China; 5 Shandong Center for Disease Control and Prevention, Jinan, China

**Keywords:** *Chlamydia trachomatis*, *Neisseria gonorrhea*, Prevalence, Risk factor, Medical cost, General population, China

## Abstract

**Background:**

A population-based study of *Chlamydia trachomatis* (CT) infections is essential in designing a specific control program; however, no large investigation of CT infections among the general population in mainland China has been conducted since 2000. We aimed to determine the prevalence, risk factors, and associated medical costs of CT among residents, 18–49 years of age, in Shandong, China.

**Methods:**

From May to August 2016, a multistage probability sampling survey involving 8074 individuals was distributed. Data were collected via face-to-face interviews, followed by self-administered questionnaire surveys. First-void urines were collected and tested for CT and *Neisseria gonorrhoeae* (NG) using nucleic acid amplification.

**Results:**

The weighted prevalence of CT infection was 2.3% (95% confidence interval [CI], 1.5–3.2) in females and 2.7% (1.6–3.8) in males. Women, 30–34 years of age, had the highest prevalence of CT infections (3.5%, 2.6–4.4), while the highest prevalence of CT infections in males was in those 18–24 years of age (4.3%, 0.0–8.8). *Neisseria gonorrhoeae* infection had a prevalence of 0.1% (0.0–0.3) in women and 0.03% (0.0–0.1) in men. Risk factors for CT infections among females included being unmarried, divorced, or widowed (odds ratio [OR], 95% CI 3.57, 1.54–8.24) and having two or more lifetime sex partners (3.72, 1.14–12.16). Among males, first intercourse before 20 years of age (1.83, 1.10–3.02) and having two or more lifetime sex partners (1.85, 1.14–3.02) were associated with CT infections. The estimated lifetime cost of CT infections in patients 18–49 years of age in Shandong was 273 million (range, 172–374 million) China Renminbi in 2016.

**Conclusions:**

This study demonstrated a high burden of CT infections among females < 35 years of age and males < 25 years of age in Shandong. Thus, a CT infection control program should focus on this population, as well as others with identified risk factors.

**Electronic supplementary material:**

The online version of this article (10.1186/s12879-018-3432-y) contains supplementary material, which is available to authorized users.

## Background

*Chlamydia trachomatis* (CT) is the most commonly diagnosed bacterial sexually transmitted infection (STI) worldwide [1]. According to the 2015 global STI surveillance report from the World Health Organization (WHO), global estimates of new CT infections in 2012 were approximately 131 million [2]. Legally notifiable STIs, such as syphilis and HIV, have increased significantly in China from 2004 to 2013, with annual percentage changes of 16.3% for both STIs [3]; however, little information is available regarding the incidence or prevalence of CT infections among the general population in mainland China over the past decade. The current estimated burden of CT infections in China is based primarily on national or regional STIs surveillance sites; however, surveillance data does not measure the true burden of CT infections among the general population because infections are often asymptomatic and undiagnosed. Furthermore, the reported data are frequently misclassified or duplicated [4, 5]. A population-based survey of STIs provided an estimate of the prevalence, determinants, and economic burden of CT infections, which facilitate the design and delivery of a CT infection control program [4]. Because the first national population-based CT infection investigation conducted in China was in 1999–2000, more than 18 years ago, the information for CT infection control and prevention strategies is outdated [6]. Another population-based CT infection survey was conducted in Shanxi Province of China in 2004, but the sample size was small (*n* = 399) [7]. Therefore, a population-based CT infection study with a large sample size is needed in China.

The Chinese government replaced the one-child policy with a universal two-child policy in October 2015, implying that more couples have opportunity to have their second child [8]. Thus, more CT infections involving couples may suffer from infertility and adverse pregnancy outcomes, such as ectopic pregnancy, without early screening [9]. The complication burden due to CT infection may be higher after the introduction of the universal two-child policy. Furthermore, CT infections and other STIs could facilitate transmission of HIV [10]. Fortunately, most of the bacterial STIs, including CT infections are treatable, so screening among high-risk populations enables early identification and treatment, which reduce the disease burden and adverse outcomes [11].

A number of population-based studies of CT infections have been conducted, including the United States and European and Oceania countries [4, 5, 12, 13]. Several strategies to improve sexual health have been implemented in a number of developed countries or regions based on findings from these population-based studies [14, 15]. Thus, it is essential to conduct a population-based CT infection investigation and provide official recommendations for CT infection-specific control interventions in China.

Shandong Province is a coastal province located in eastern China, with a population of 98.5 million in 2015, accounting for 7% of the total population in China. This study was a population-based, cross-sectional study conducted from 9 May to 25 August 2016 in Shandong Province. We undertook this study to determine the prevalence, risk factors, and associated medical costs of CT infections among the general population 18–49 years of age in China.

## Methods

### Study design and participants

**Fig. 1:**
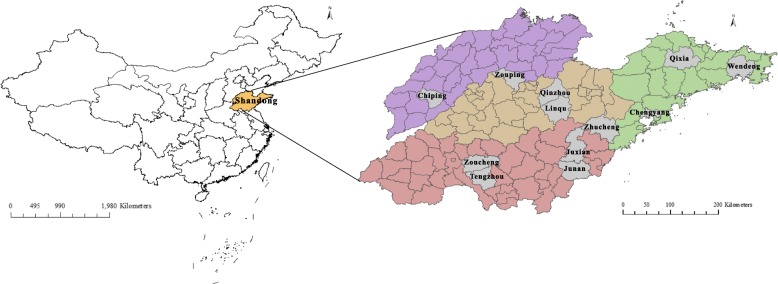
Geographic locations of Shandong Province and 12 primary sampling units

The population was sampled to represent the general population of Shandong Province, China (18–49 years of age). General population refers to males and females who had lived in their current residence for at least the past 6 months. The estimated sample size was 8074 individuals based on the national survey conducted in 1999–2000 [6]. (Additional file 1) The multi-stage sampling process included four steps, as follows: Shandong Province was divided into four strata based on geographic region (east, northwest, south, and middle); 2–4 urban districts or counties were randomly extracted from each stratum, producing 12 primary sampling units (PSUs; Fig. 1); 4–7 subunits (rural townships and urban street districts) were selected from each PSU using the probability proportionate to size sampling (PPS) method; a total of 59 subunits were selected; 3–12 rural villages or urban communities were selected from each subunit using the aforementioned sampling method, with more populated subunits having a higher probability of selection; villages and communities were selected with a proportion of 1:1; a total of 184 rural villages and 183 urban communities were included; residents, 18–49 years of age, who had lived in their current residence for at least the past 6 months were selected and arrayed in order by age and gender; systematic sampling was performed based on the gender and age distribution of the Shandong Province population to produce the sample; Thus, 22 persons were drawn from each village or community according to sample size and number of selected villages or communities.

The inclusion criteria were as follows: participants born between 1 January 1966 and 31 September 1997; continuous residence at the study site for at least the past 6 months; consent to provide urine specimens for testing; and willing to participate in the study and complete the questionnaires. The exclusion criteria were as follows: individuals denying sexual debut (including oral sex); and inability to provide correct information, such as individuals with a mental illness or alcohol abusers.

This study was approved by the Ethics Committee of Shandong Provincial Institute of Dermatology and Venereology (approval number: 2016–04). Oral informed consent was obtained from each participant by interviewers before interview.

### Procedures

Before implementation of this survey, 3 days of training on fieldwork staff was provided by epidemiology and clinical laboratory professionals in each of the PSUs. One laboratory technician for urine collection and coding, one male and one female questionnaire interviewers, and one financial staff for providing rewards to participants were selected on the basis of course performance for each field team. A trained supervisor from the study group was also assigned to each field team for ethics and quality control of the interview.

Before sampling, individuals who continuously lived in their current residences for at least the past 6 months were identified by village/community committee staff or physicians by 3 steps. First, they referred to the official registers of household or health record of local residents for eligible individuals. Second, they inquired and recorded unregistered migrants who had lived in the local village/community for 6 months or more. Third, these two groups of people were combined in a roster for sampling. The sampled participants were informed to be interviewed in the village/community clinic or meeting room in private. For individuals who could not be reached via telephone, staff or physicians would visit them in their home. Individuals who could not be reached after repeated visits were excluded from the study. To retain privacy, questionnaire interviews were performed in a private room, and interviewers were the same gender as the respondent. Most of the selected respondents worked during the daytime, so the interviews were usually performed early in the morning or in the evening. Computer-based interviews were not performed because the pilot investigation showed that many participants could not use the computer to navigate the questionnaire, especially in villages. We adopted questionnaire-based interviews to collect information. Face-to-face interviews addressed demographic information, including gender, age, marital status (unmarried, married, divorced, or widowed), educational level (elementary school or below, middle school, senior high school or technical school, and university or above), and location of residence (rural or urban). Other information on the questionnaires was completed by the respondents themselves. The structured questionnaires included information regarding occupation, income, living status (living with family, living with roommates, or living alone), living with a spouse (yes or no), smoking (a smoker was defined as someone who had smoked > 100 cigarettes during their lifetime), number of episodes of intoxication in the past year (0, 1–3, 4–6, and > 6), STIs (including syphilis, gonorrhea, CT infection, condyloma acuminatum, genital herpes, and HIV infection) in the past 5 years (yes or no), quality of sex life (satisfied or not satisfied), sexual behaviors, such as age at first intercourse (≤20 years and > 20 years), number of sex partners (1 and ≥ 2), and number of new sex partners in the past year (≤ 1 and ≥ 2). After the interview, the supervisor checked the questionnaires for missing values and logical errors and wrote a unique alphanumeric code on the questionnaires instead of the individual’s name.

Every respondent was instructed not to urinate for at least 1 h before they participated in the interview. Approximately 30–50 ml of first-void urine was collected into a urine cup. Laboratory technicians promptly transferred the urine into Cobas PCR Media tubes (Roche Molecular Systems, Inc., Branchburg, New Jersey, USA) using a disposable pipette and the tube was inverted 5 times to mix. Each specimen was labeled with a unique alphanumeric code, which was the same as on the questionnaire. Urine was transported inside a Styrofoam cooler to the local laboratory within 3 h of collection and was stored 4 °C as soon as the staff received the specimen. Urine was transported inside the cooler to the laboratory of Shandong Provincial Institute of Dermatology and Venereology in Jinan within 2 weeks.

*Chlamydia trachomatis* and *Neisseria gonorrhoeae* (NG) DNA in urine specimens were detected with a Roche Cobas 4800 CT/NG Assay (Roche Molecular Systems, Inc.), which had a sensitivity of 96.6% and a specificity of 100% [16]. Only participants with positive results were notified, then referred to a local STI clinic or general hospital for treatment.

### Statistical analysis

Questionnaire information, as well as testing results, was double-entered into Epidata 3.1 (EpiData association, Odense, Denmark); discrepancies were checked against raw data. We applied selection probability weights which were inversely proportional to the selection probabilities for the number of units at each sampling step to balance differences in the probabilities of selection. Non-response weighting was made by adjustment among those who participated in this survey with those who did not. Post-stratification weighting was made by adjusting data according to age and gender distribution of the Shandong census data. We analyzed data with *surveyfreq* and *surveylogistic* methods using SAS 9.3 (SAS Institute, Inc., Cary, NC, USA). The prevalence with 95% confidence interval (CI) of CT and NG was calculated based on total population weights and Taylor series linearization. We determined the association between CT infection and demographic and behavioral variables with logistic regression and reported crude odds ratio (ORs) in univariate analysis. Risk factors with a *p* value < 0.20 in univariate analysis were applied into the multivariate logistic model. Adjusted ORs and 95% CIs are presented. The variance inflation factor (VIF), condition index, and variance proportions were calculated to examine multicollinearity among risk factors. Maximal variance inflation factor values ≥10 or maximal condition index values ≥10 and a corresponding variance proportion value > 0.5 indicated multicollinearity among variables. All tests were two-sided, and a *p* value < 0.05 was considered statistically significant.

**Fig. 2:**
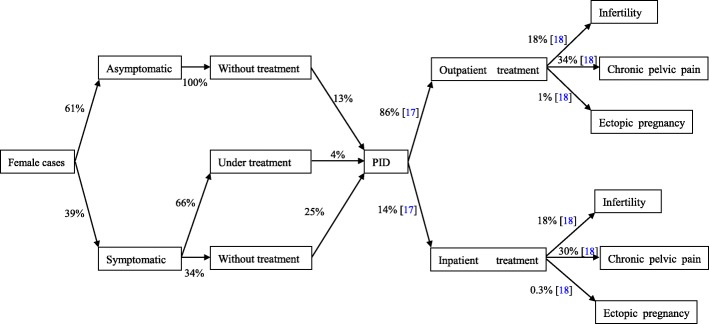
The method of calculating the medical costs of CT infection for females. *Abbreviations*: *PID* pelvic inflammatory disease, *CT Chlamydia trachomatis*

**Fig. 3:**
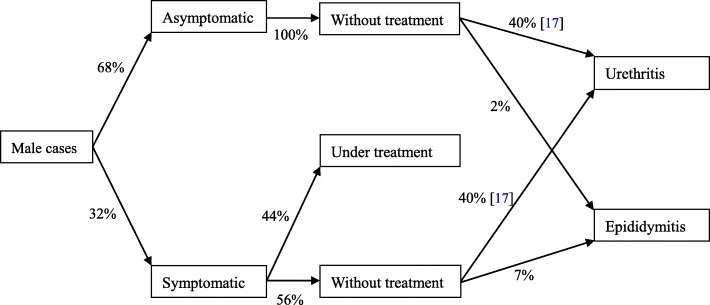
The method of calculating the medical costs of CT infection for males. *Abbreviations*: *CT Chlamydia trachomatis*

Proportion of symptomatic and treatment, incidences of various complications after CT infection were on the basis of our primary data as well as previous published studies [17, 18] (Figs. 2 and 3, Additional file 2). Medical costs for CT infections were based on diagnosis and treatment of the infection and complications; costs related to transportation, lodging, and time missed from work were not included into analysis. According to guidelines for STI treatment and control in China, we assumed that the diagnosis of CT infections was established with PCR testing and the treatment of CT infections involved a single 1 g dose of oral azithromycin [19]. Medical costs of cases with complications were estimated based on published research in Shandong Province [17]. The discounted rate of medical costs was assumed to be 3%. Sensitivity analysis was performed by adding uncertainty factors, such as the prevalence of CT infections, proportion of asymptomatic CT infections [20], and diagnostic method (antigen detection), into the computation.

## Results

**Fig. 4:**
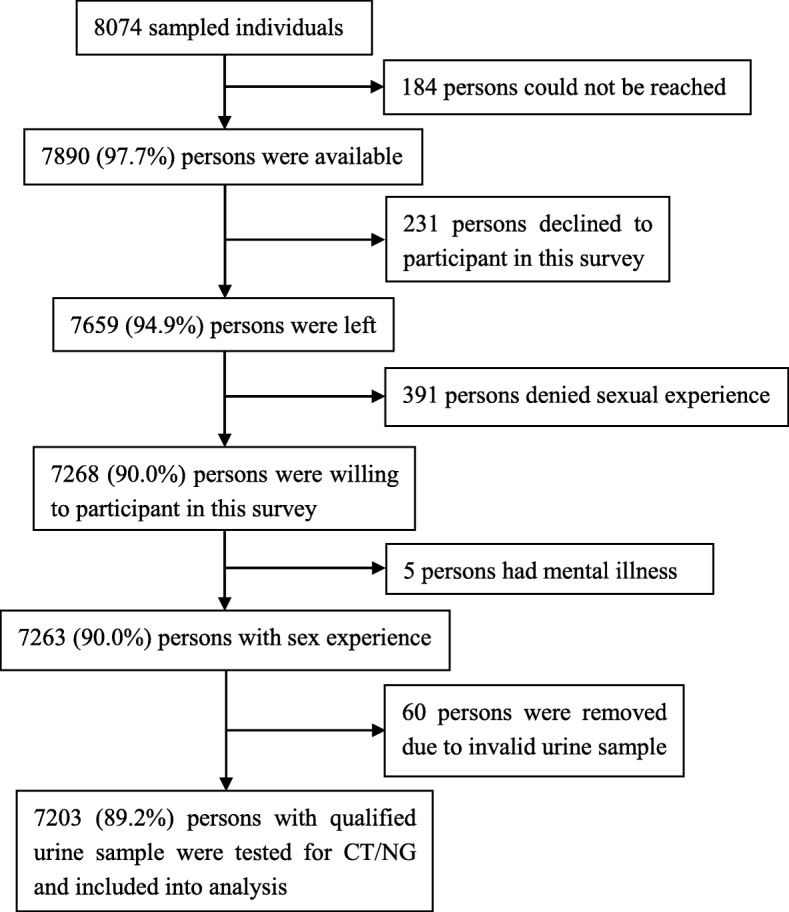
Flow chart for participant selection. *Abbreviations*: *CT Chlamydia trachomatis*, *NG neisseria gonorrhoeae*

Of the 8074 individuals originally sampled, 184 could not be reached via telephone and were not at home on repeated visits. Among the 7890 individuals who were contacted, 231 declined to participate in this survey, 391 denied a sexual debut, and 5 had mental illnesses, leaving 7263 (90.0%) individuals who participated in the study and provided a urine sample. In addition, 60 urine samples were excluded due to mislabeling or insufficient urine, leaving 7203 (89.2%) satisfactory urine sample with test results for analysis (Fig. 4).

### Prevalence of CT and NG infections

The weighted age-specific prevalence of CT and NG infections is shown in Table 1. Of the 7203 urine samples tested, 167 participants (90 females and 77 males) tested positive for CT. The estimated prevalence of CT in the general population, 18–49 years of age, in 2016 was 2.3% (95% CI, 1.5–3.2) for females and 2.7% (95% CI, 1.6–3.8) for males, corresponding to approximately 1,263,000 infections (95% CI, 774,000-1,753,000) in Shandong. No significant difference in the prevalence of CT infections existed between females and males (*p* = 0.52). The highest prevalence of CT infections occurred in females 30–34 years of age (3.5%; 95% CI, 2.6–4.4), followed by females 18–24 (3.2%; 95% CI, 0.4–6.1) and 25–29 years of age (2.9%; 95% CI, 1.9–3.8). The prevalence of CT infections in females 18–34 years of age (3.2%; 95% CI, 1.8–4.6) was significantly higher than females 35–49 years of age (1.5%; 95% CI, 0.8–2.2 [OR, 2.16; 95% CI, 1.14–4.08]); however, the highest prevalence of CT infections occurred in males 18–24 years of age (4.3%; 95% CI, 0.0–8.8). For females, the highest prevalence was in those from South region (3.0%, 95%CI, 2.2–3.9) of Shandong. By contrast, for males, the highest prevalence was in those from Northwest (3.0%, 95%CI, 0.0–7.7) and Middle region (3.0%, 95%CI, 1.1–4.9) of Shandong. The prevalence of CT infections for urban and rural residents was 2.8% (95% CI, 1.5–4.0) and 2.2% (95% CI, 1.4–2.9), respectively, but the difference was not statistically significant (*p* = 0.42).

**Table 1:** The weighted prevalence of CT and NG in participants 18–49 years of age in Shandong, China

	Male [n, %(95%CI)]		Female [n, %(95%CI)]		Denominator^a^ (unweighted, weighted^b^)	
	CT	NG	CT	NG	Male	Female
Age(years)						
18–24	11, 4.3% (0.0–8.8)	0, 0.0%	7, 3.2% (0.4–6.1)	1, 0.2% (0.0–0.6)	253, 851	194, 825
25–29	18, 2.3% (0.5–4.1)	0, 0.0%	24, 2.9% (1.9–3.8)	0, 0.0%	780, 496	914, 499
30–34	13, 2.8% (1.3–4.2)	0, 0.0%	20, 3.5% (2.6–4.4)	1, 0.1% (0.0–0.2)	500, 469	555, 461
35–39	13, 2.7% (1.1–4.2)	0, 0.0%	8, 1.4% (0.4–2.4)	1, 0.1% (0.0–0.3)	557, 567	588, 559
40–44	15, 2.4% (1.3–3.5)	0, 0.0%	14, 1.5% (0.4–2.6)	1, 0.1% (0.0–0.4)	713, 667	735, 659
45–49	7, 0.8% (0.2–1.3)	1, 0.2% (0.0–0.5)	17, 1.6% (0.9–2.4)	1, 0.1% (0.0–0.3)	692, 572	722, 578
Region						
Northwest	4, 3.0% (0.0–7.7)	0, 0.0%	7, 1.6% (0.6–2.5)	0, 0.0%	338, 390	348, 356
Middle	28, 3.0% (1.1–4.9)	0, 0.0%	17, 2.0% (0.0–4.6)	0, 0.0%	895, 941	907, 851
South	29, 2.7% (1.7–3.8)	1, 0.1% (0.0–0.2)	46, 3.0% (2.2–3.9)	2, 0.1% (0.0–0.5)	1489, 1456	1567, 1479
East	16, 2.0% (1.3–2.7)	0, 0.0%	20, 1.8% (1.1–2.5)	3, 0.2% (0.0–0.5)	773, 835	886, 895
Total	77, 2.7% (1.6–3.8)	1, 0.03% (0.0–0.1)	90, 2.3% (1.5–3.2)	5, 0.1% (0.0–0.3)	3495, 3622	3708, 3581

*Abbreviations: CI* confidence interval, *CT* chlamydia trachomatis, *NG* neisseria gonorrhoeae

^a^Denominator was participants with sex experience and a urine test result

^b^Selection probability weighting, non-response weighting, and post-stratification weighting were applied to calculate the weighted denominator

Of the 7203 urine samples tested, 6 participants (5 females and 1 male) tested positive for gonorrhoea. The estimated prevalence of gonorrhoea in the target population in 2016 was 0.1% (95% CI, 0.0–0.3) for females and 0.03% (95% CI, 0.0–0.1) for males, but the difference was not significant between female and male residents (*p* = 0.25). The estimated number of NG infections in Shandong corresponded to approximately 35,000 cases (95% CI, 0–83,000). The prevalence of NG infections among urban residents (0.10%; 95% CI, 0.0–0.2) was significantly higher than rural residents (0.03%; 95% CI, 0.0–0.1 [OR, 3.58; 95% CI, 1.08–11.83]). Because of the limited number of NG-positive cases, the following analysis focused on CT infections.

### Risk factors for CT infections

Based on univariate analyses, risk variables associated with CT infections for both males and females 18–49 years of age included being unmarried/divorced/widowed, first sexual intercourse before 20 years of age, and having ≥2 sex partners. For females, an increased prevalence of CT infections was also associated with a higher level of education. For males, an increased prevalence of CT infections was associated with living alone, living separated from their spouse, and having been infected with a STI in the past 5 years, while a decreased prevalence of CT infections was associated with increasing age (Table 2).

**Table 2:** Risk factors for CT in urine specimens from participants 18–49 years of age in Shandong, China

	Male				Female				Denominator^c^ unweighted, weighted	
	% (95% CI)	Crude OR	Adjusted OR^a^	95% CI	% (95% CI)	Crude OR	Adjusted OR^b^	95% CI	Male	Female
All ages	2.7% (1.6–3.8)				2.3% (1.5–3.2)				3495, 3622	3708, 3581
Age(years)		*P* = 0.02	*P* = 0.48			*P* = 0.08	*P* = 0.57			
18–24	4.3% (0.0–8.8)	1.00	1.00	1.00–1.00	3.2% (0.4–6.1)	1.00	1.00	1.00–1.00	253, 851	194, 825
25–29	2.3% (0.5–4.1)	0.52	0.91	0.24–3.48	2.9% (1.9–3.8)	0.88	2.23	0.72–6.86	780, 496	914, 499
30–34	2.8% (1.3–4.2)	0.63	1.40	0.37–5.35	3.5% (2.6–4.4)	1.08	3.74	1.09–12.83	500, 469	555, 461
35–39	2.7% (1.1–4.2)	0.61	1.40	0.37–5.21	1.4% (0.4–2.4)	0.42	1.54	0.58–4.06	557, 567	588, 559
40–44	2.4% (1.3–3.5)	0.54	1.32	0.54–3.24	1.5% (0.4–2.6)	0.46	1.48	0.45–4.86	713, 667	735, 659
45–49	0.8% (0.2–1.3)	0.17	0.40	0.15–1.03	1.6% (0.9–2.4)	0.50	1.81	0.60–5.45	692, 572	722, 578
Education		*P* = 0.70				*P* = 0.02	*P* = 0.07			
Elementary school, or below	3.2% (0.0–8.3)	1.00	–	–	1.6% (1.1–2.2)	1.00	1.00	1.00–1.00	367, 330	806, 697
Middle school	2.5% (1.4–3.5)	0.75	–	–	1.8% (1.2–2.3)	1.08	1.04	0.69–1.55	1906, 1830	1935, 1788
Senior high school or technical school	3.3% (1.6–5.0)	1.02	–	–	3.1% (0.8–5.3)	1.90	1.52	0.80–2.92	920, 1040	669, 752
College, university, or above	1.4% (0.0–3.1)	0.44	–	–	5.2% (0.0–10.6)	3.32	2.11	0.93–4.81	302, 422	298, 344
Marital status		*P* = 0.01	*P* = 0.45			*P* < 0.01	*P* < 0.01			
Married	2.1% (1.5–2.8)	1.00	1.00	1.00–1.00	1.8% (1.2–2.4)	1.00	1.00	1.00–1.00	3164, 3043	3528, 3253
Unmarried, divorced or widowed	5.4% (0.9–9.9)	2.61	1.45	0.55–3.84	7.8% (1.4–14.3)	4.65	3.64	1.63–8.13	331, 579	180, 328
Location of residence		*P* = 0.80				*P* = 0.22				
urban	2.8% (1.1–4.5)	1.00	–	–	2.8% (1.0–4.5)	1.00	–	–	1708, 2063	1824, 2040
rural	2.5% (1.2–3.9)	0.91	–	–	1.8% (1.3–2.2)	0.64	–	–	1787, 1559	1884, 3581
Living status		*P* = 0.01	*P* = 0.17			*P* = 0.75				
living with family	2.3% (1.5–3.1)	1.00	1.00	1.00–1.00	2.3% (1.4–3.2)	1.00	–	–	3279, 3327	3538, 3320
living with roommates	6.5% (0.0–17.8)	2.99	2.20	0.53–9.19	2.9% (0.0–9.3)	1.29	–	–	69, 99	54, 108
living alone	7.3% (0.0–16.4)	3.38	1.77	0.50–6.22	2.8% (0.0–6.0)	1.21	–	–	147, 196	116, 153
Living with spouse^d^		*P* = 0.01				*P* = 0.92				
Yes	1.9% (1.5–2.3)	1.00	–	–	1.8% (1.1–2.5)	1.00	–	–	3080, 3149	3309, 3213
No	10.4% (0.0–24.6)	5.93	–	–	1.9% (0.3–3.5)	1.06	–	–	84, 86	219, 244
Smoking		*P* = 0.18	*P* = 0.42			*P* = 0.31				
No	2.1% (0.9–3.3)	1.00	1.00	1.00–1.00	2.3% (1.5–3.1)	1.00	–	–	1747, 1785	3309, 3527
Yes	3.2% (1.5–4.9)	1.58	1.36	0.65–2.86	5.9% (0.0–16.5)	2.68	–	–	1748, 1837	70, 54
STI in the past 5 years^e^		*P* = 0.01	*P* = 0.08			*P* = 0.19	*P* = 0.13			
No	2.5% (1.4–3.6)	1.00	1.00	1.00–1.00	2.3% (1.5–3.1)	1.00	1.00	1.00–1.00	3467, 3595	3697, 3572
Yes	21.1% (0.0–54.8)	10.3	7.65	0.79–73.84	9.0% (0.0–28.4)	4.15	6.21	0.58–66.18	19, 27	11, 9
Age at first intercourse (years)		*P* = 0.01	*P* = 0.03			*P* < 0.01	*P* = 0.24			
>20	1.8% (1.4–2.3)	1.00	1.00	1.00–1.00	1.9% (1.2–2.6)	1.00	1.00	1.00–1.00	2698, 2549	3308, 3000
≤ 20	4.6% (1.0–8.2)	2.58	1.76	1.05–2.94	4.7% (2.5–7.0)	2.59	1.94	0.64–5.90	797, 1073	400, 581
Number of sex partners		*P* < 0.01	*P* = 0.02			*P* < 0.01	*P* = 0.04			
1	2.1% (1.5–2.8)	1.00	1.00	1.00–1.00	2.0% (1.2–2.7)	1.00	1.00	1.00–1.00	3086, 3107	3597, 3441
≥ 2	6.0% (1.5–10.4)	2.92	2.09	1.10–3.94	11.5% (0.0–23.3)	6.47	3.66	1.07–12.48	409, 515	111, 140
Number of new sex partners in the past year		*P* = 0.14	*P* = 0.40			*P* = 0.44				
≤ 1	2.5% (1.4–3.7)	1.00	1.00	1.00–1.00	2.3% (1.5–3.1)	1.00	–	–	3375, 3459	3678, 3549
≥ 2	5.2% (0.5–9.8)	2.08	0.56	0.15–2.16	5.0% (0.0–12.0)	2.21	–	–	120, 163	30, 32

*Abbreviations*: *CT* Chlamydia trachomatis, *CI* confidence interval, *OR* odds ratio, *AOR* adjusted odds ratio, *STI* sexually transmitted infection

^a^Adjusted for age, marital status, living status, smoking, STI in the past 5 years, age at first intercourse, number of sex partners, number of new sex partners in the past year and smoking

^b^Adjusted for age, education, marital status, STI in the past 5 years, age at first intercourse and number of sex partners

^c^Denominator was participants with sex experience and a urine test result

^d^This variable included those who have been married

^e^STIs included syphilis, gonorrhea, chlamydia trachomatis infection, condyloma acuminatum, genital herpes, and HIV infection

In the multivariate logistic model, factors remaining significantly associated with CT infection for women were being unmarried/divorced/widowed (OR, 3.57; 95% CI, 1.54–8.24) and having ≥2 sex partners (OR, 3.72; 95% CI, 1.14–12.16). A higher level of education was marginally associated with CT infections in females. In males, first sexual intercourse before 20 years of age (OR, 1.83; 95% CI, 1.10–3.02) and having ≥2 sex partners (OR, 1.85; 95% CI, 1.14–3.02) remained significantly associated with CT infections (Table 2).

In the collinearity diagnosis, the maximal VIF was 1.17 and the maximal condition index was 1.55 for the female regression model, while the maximal VIF was 1.29 and the maximal condition index was 1.87 for the male regression model, which indicated no multicollinearity in the multivariate logistic analysis.

### Medical costs for CT infections

Based on calculated costs for treatment of CT complications in 2016 (Additional file 2), we estimated that the lifetime total costs for CT infections for patients 18–49 years of age in Shandong Province was 273 million Renminbi (RMB), which corresponds to 348 RMB for each CT infection in females and 102 RMB for each CT infection in males.

In sensitivity analysis, the estimated range for the total lifetime costs of CT infections in patients 18–49 years of age in Shandong was 172–374 million RMB in 2016. The estimated range of medical costs for each female and male patient was 339–361 RMB and 97–108 RMB, respectively. (Additional file 2).

## Discussion

This is the first representative population-based cross-sectional study with a large sample size to determine the epidemiology of CT infections in mainland China since 1999–2000. In addition, the medical costs of CT infections and associated complications were estimated for the first time in China. Compared with the only national population-based STI prevalence study conducted in China (1999–2000), which reported a CT prevalence of 0.6% for females and 1.1% for males 20–24 years of age in China, the results from this study showed that the prevalence of CT infections has now increased 5-fold for females and 3-fold for males in this age group [6]. This change appears to be influenced by increased premarital and extramarital sexual activity in China, especially among youth [21]. According to a study conducted in 1999, approximately 15% of male and 9% of female university students had participated in premarital sexual activity [22]; however, another study concluded that 26% of college students had sexual intercourse in 2010 [21]. The rate of premarital sexual activity was even higher among out of school/community young adults than among those in school [23].

We compared our study with other population-based cross-sectional surveys that have used a complex sampling design and urine tests of infection. In 2014–2016, Wong WC et al. performed a survey of 733 sexually experienced individuals in Hong Kong, China [24]. Among females 18–49 years of age, prevalence of CT infections was 2.0% (95% CI, 1.0–3.7) in Hong Kong compared with 2.3% (95% CI, 1.5–3.2) in Shandong Province. Among males 18–49 years of age, the prevalence was 1.5% (95% CI, 0.6–3.6) in Hong Kong compared with 2.7% (95% CI, 1.6–3.8) in Shandong. No significant difference of CT prevalence was found between these two studies due to overlap of 95% CI. The third cycle of the National Surveys of Sexual Attitudes and Lifestyles (Natsal-3), which was conducted in the UK in 2010–2012, indicated that the prevalence of CT infections was 1.5% (95% CI, 1.1–2.0) and 1.1% (95% CI, 0.7–1.6) for females and males 16–44 years of age, respectively [4]. In this study, prevalence of CT among males 18–44 years of age (3.0%, 95% CI, 1.8–4.3) was nearly 2 times higher than that in the UK, while difference of CT prevalence for females 18–44 years of age (2.5%, 95% CI, 1.5–3.4) was not significantly. Data from the 2011–2012 American National Health and Nutrition Examination Survey (NHANES) showed that the prevalence of CT infections among females and males 14–39 years of age was 2.0% (95% CI, 1.5–2.5) and 1.4% (95% CI, 1.1–1.8), respectively [25]. The difference of CT prevalence between males 18–39 years of age (3.2%, 95% CI, 1.8–4.6) in our study and that in the United States was marginally significant, while prevalence of CT for females 18–39 years of age (2.8%, 95% CI, 1.6–3.9) was as high as that in the US. Therefore, males in Shandong Province may suffer a higher burden of CT infection than that in the UK and US.

Our study also indicated that CT infections were more prevalent among females < 35 years of age and males < 25 years of age. Thus, these young adults need to be prioritized when implementing a CT infection control program. A previous study showed that screening for CT infections is cost-effective when the prevalence of CT infections among the female population ranged from 3.1 to 10.0% [26]. This finding indicated that the female population < 35 years of age in Shandong should be screened regularly for CT infections; however, the effectiveness of CT screening to control transmission and prevent complications of CT infection is controversial. In the UK, The National Chlamydia Screening Programme (NCSP), implemented in September 2002, offers opportunistic screening for CT infections among males and females < 25 years of age [15]; however, according to the Natsal-2 (conducted in 1999–2000) compared with Natsal-3, the prevalence of CT infections in young people 18–24 years of age was similar in females (3.1% vs. 3.2%), and males (2.9% vs. 2.6%) [4]. In 2001, the United States Preventive Services Task Force (USPSTF) recommended that clinicians routinely screen all sexually-active females ≤25 years of age, and other asymptomatic females at increased risk for CT infections [27]; however, NHANES reported a similar prevalence of CT infections in the 1999–2000 (4.1%) and 2007–2008 cycles (3.8%) among females 14–25 years of age [28]. In addition, randomized controlled trials (RCTs) also revealed contradictory evidence on the effectiveness of a CT infection screening strategy [29, 30]. The low screening efficacy on reducing the prevalence of CT infections and associated complications may be due to several factors. First, the uptake rate of CT screening is quite low in most countries that conduct screening programs. In 2017, an estimated 28% of young females and 11% of young males were tested for CT infections in England [31]. In the US, the estimated annual CT testing rate for sexually-active females 15–25 years of age was 37.9% [32]. Second, annual screening may not be frequent enough to prevent complications of CT infections. A RCT from the UK indicated that PID over 12 months was not prevented by a single CT screen and occurred in women who were negative for CT at baseline [30]. Thus, it is critical to answer questions, such as how to increase screening coverage and how often to screen through RCT or other type of studies before performing universal CT screening in Shandong, China. In our study, women 30–34 years of age had the highest prevalence of CT infections, while the highest prevalence of CT infections in males was in those 18–24 years of age. The age differentials of CT prevalence may be associated with variation of sexual desire. For males, sexual desire significantly increased after puberty and reached its peak at about 18–30 years of age [33]. For females, the sexual desire reached its peak at around 30–40 years of age [33]. Strong desire usually accompanied with frequent sexual activity, which may contribute to the high prevalence of STIs, including CT infections.

A multivariate analysis indicated that younger age at the time of first sexual intercourse is associated with a higher risk of CT infections in men. Another study showed the same conclusion among adolescents and young adults [34]. Early initiation of sexual intercourse was shown to be associated with multiple partners, more casual or commercial sex partners, and infrequent use of condoms, resulting in a higher prevalence of STIs [35]. Therefore, sex education strategies should focus on an earlier age, advice on delaying the age of first sexual intercourse, and address other issues, such as reducing the number of partners and promoting condom use [35]. The prevalence of CT infections among unmarried, divorced, or widowed females 18–49 years of age was 7.8%, which was 4 times the prevalence among married counterparts (1.8%) in this study. Data from the NHANES in 2007–2012 showed that the risk of CT infection among divorced/widowed individuals was 3 times higher than married persons [25]. Marital status is therefore a useful marker for identifying those at increased risk for a CT infection. Compared with the national CT study in 1999–2000, the difference in prevalence of CT infections among urban and rural residents is not statistically significant, which may be due to increasing proportion of migrants among the general population in China [6, 36]. The prevalence of CT was much higher for migrants than the non-migrant population [37]. Frequently, the flow of a large number of migrants may decrease the difference in STIs between urban and rural populations.

The results of this study indicate that CT infections produce a substantial economic burden in Shandong Province. The total medical costs of CT infections in Shandong Province are mainly influenced by the variation in the prevalence of CT infections rather than different methods of detection. Thus, adequate public health financial support is required to support specific CT control strategies.

Our study had several limitations. First, individuals who left their original place of residence and had lived in their current residence for ≥6 months were not completely registered by the local government and it was difficult to select all of them for sampling; however, this unregistered population could engage in more risky behaviors leading to STIs [6], thus resulting in an underestimation of the prevalence of CT and NG in our study. Second, our estimate of economic burden of CT was partially based on previous surveys. The incidence of complications after CT infection in other populations may not precisely correspond with that in Shandong Province, China. Third, the conclusions of this study might not be applicable to all provinces in China. Fourth, the statistical power of this study might be insufficient to identify potential risk factors of CT infection that were poisson distributed among general population, such as men who have sex with men (MSM) or female sex worker (FSW).

## Conclusions

In conclusion, the results indicate that the prevalence of CT infections in young adults has increased rapidly since 2000. *Chlamydia trachomatis* is highly prevalent among females < 35 years of age and males < 25 years of age. Thus, CT control programs should focus on this population, as well as other people with identified risk factors, to reduce the prevalence and economic burden of CT infections. Further studies evaluating the effectiveness of CT control interventions through RCTs or other types of study design in China are recommended.

## Additional files

Additional file 1: Sample size calculation process. (DOCX 12 kb)

Additional file 2: Estimation process for medical cost of CT infection. (DOCX 20 kb)

## Abbreviations

CI: Confidence interval
CT: Chlamydia trachomatis
FSW: Female sex worker
MSM: Men who have sex with men
Natsal: National surveys of sexual attitudes and lifestyles
NCSP: National chlamydia screening programme
NG: Neisseria gonorrhoeae
NHANES: National health and nutrition examination survey
OR: Odds ratio
PID: Pelvic inflammatory disease
PPS: Probability proportionate to size sampling
PSU: Primary sampling unit
RCT: Randomized controlled trial
RMB: Renminbi
STI: Sexually transmitted infection
USPSTF: United States preventive services task force
VIF: Variance inflation factor
WHO: World health organization
