# Supplementary material for: Prevalence, risk factors, and medical costs of Chlamydia trachomatis infections in Shandong Province, China: a population-based, cross-sectional study
Source: BMC Infect Dis. 2018 Oct 26;18:534. doi: 10.1186/s12879-018-3432-y (PMC6204023; doi:10.1186/s12879-018-3432-y)
Supplement: Supplementary file 1 — Sample size calculation process. (DOCX 12 kb) [file 12879_2018_3432_MOESM1_ESM.docx]

**Additional file 1: Sample size calculation process**

We assumed that the prevalence of CT infections among participants was 3.29% based on the national survey conducted in 1999-2000 [6]. The assumed allowable error was 0.66%, which was 0.2 times the assumed prevalence of CT infections. The type I error was set at 0.05 and the non-response rate was assumed to be 30%. Design effect refers to the ratio of actual variance of this study design and variance of a simple random sampling design with the same sample size, which was assumed to be 2. Based on these assumptions, the estimated sample size was 8068 individuals.

A total of 184 rural villages and 183 urban communities were sampled in this study. To avoid decimals, the sample size was increased from 8068 to 8074; thus, 22 persons were drawn from each village or community.
