# Supplementary material for: Prevalence, risk factors, and medical costs of Chlamydia trachomatis infections in Shandong Province, China: a population-based, cross-sectional study
Source: BMC Infect Dis. 2018 Oct 26;18:534. doi: 10.1186/s12879-018-3432-y (PMC6204023; doi:10.1186/s12879-018-3432-y)
Supplement: Supplementary file 2 — Estimation process for medical cost of CT infection. (DOCX 20 kb) [file 12879_2018_3432_MOESM2_ESM.docx]

**Additional file 2: Estimation process for medical cost of CT infection**

**1. Assumed proportion of symptomatic and treatment, and assumed incidences of various complications after *Chlamydia trachomatis* infection**

Ninety females tested positive for Chlamydia trachomatis (CT) in this study, among which 61% (55/90) was asymptomatic. None of the 55 cases were ever treated. Seven (13%) of the 55 cases were diagnosed as pelvic inflammatory disease (PID) before. Among the 35 (39%) symptomatic female cases, 23 (66%) were ever treated. One case (4%) was diagnosed as PID among the 23 treated cases while 3 (25%) PID cases were identified among 12 untreated cases (Fig. 2).

Seventy-seven males tested positive for CT, among which 68% (52/77) was asymptomatic. None of the 52 cases were ever treated too. One (2%) of the 52 cases was diagnosed as epididymitis before. Among the 25 (32%) symptomatic male cases, 11 (44%) were ever treated. One (7%) of the 14 untreated cases was diagnosed as epididymitis while no epididymitis case was identified in the 11 treated cases (Fig. 3).

Of the women with PID, 86% were assumed to be treated in an outpatient clinic, while 14% required inpatient treatment [17]. Of the outpatient treated females with PID, it was assumed that 18% would develop infertility, 34% would develop chronic pelvic pain, and 1% would develop ectopic pregnancies [18]. In contrast, it was assumed that among females with PID who were hospitalized, 18% would develop infertility, 30% would develop chronic pelvic pain, and 0.3% would develop ectopic pregnancies [18]. For untreated males with CT infections, we assumed 40% would develop urethritis [17].

**2. Calculated costs for treatment of *Chlamydia trachomatis* and complications in 2016**

According to guidelines for STI treatment in China [19], we assumed that the diagnosis of CT infections was established with PCR testing and the treatment of CT infections involved a single 1 g dose of oral azithromycin. Thus, the calculated medical costs for diagnosis and treatment of CT infections were 120 RMB in 2016 (Table S1).

For females, the calculated costs for treatment of infertility, ectopic pregnancies, chronic pelvic pain, and outpatient and inpatient PID were 3,107 RMB, 6,667 RMB, 2,118 RMB, 1,053 RMB, and 2,446 RMB, respectively, in 2016 (Table S1). For males, the calculated costs for treatment of urethritis and epididymitis were 212 RMB and 454 RMB, respectively, in 2016 (Table S1).

**Table S1** Cost items in calculation process

| **Items** | **Cost in 2002 [**17**]** | **Cost in 2016**^a^ |
| --- | --- | --- |
| Diagnosis of CT infection (PCR testing) ^b^ |  | 80 RMB |
| Diagnosis of CT infection (Antigen detection) ^b^ |  | 45 RMB |
| Treatment of CT infection (1 g azithromycin) ^b^ |  | 40 RMB |
| Treatment of infertility | 2,054 RMB | 3,107 RMB |
| Treatment of ectopic pregnancies | 4,408 RMB | 6,667 RMB |
| Treatment of chronic pelvic pain | 1,400 RMB | 2,118 RMB |
| Outpatient PID treatment | 696 RMB | 1,053 RMB |
| Inpatient PID treatment | 1,617 RMB | 2,446 RMB |
| Treatment of urethritis | 140 RMB | 212 RMB |
| Treatment of epididymitis | 300 RMB | 454 RMB |

^a^ The discounted rate of medical costs was assumed to be 3%.

^b^ Cost of diagnosis and treatment of CT infection was derived directly from that in Shandong Provincial Dermatology Hospital in 2016.

*Abbreviations*: *CT* Chlamydia trachomatis, *PCR* polymerase chain reaction, *PID* pelvic inflammatory disease, *RMB* Renminbi

**3. Assumed prevalence of *Chlamydia trachomatis* infections, proportion of asymptomatic *Chlamydia trachomatis* infections, and diagnostic method in sensitivity analysis**

When the prevalence of CT infections was at a lower level of the 95% CI, the estimated lifetime total medical costs for CT infections in patients 18-49 years of age in Shandong was 172 million RMB (Table S2). When the prevalence of CT infections was at a higher level of the 95% CI, the estimated lifetime medical costs of the cases were 374 million RMB (Table S2). The variation in the prevalence of CT infections did not influence medical costs for each CT infection. If 90% of the individuals infected with CT were asymptomatic [20], the estimated lifetime total medical costs of CT infections in patients 18-49 years of age in Shandong was 285 million RMB, which corresponded to the cost for each female and male infection of 361 RMB and 108 RMB, respectively (Table S2). If the diagnosis of CT infections was based on antigen detection instead of PCR, the medical costs for diagnosis of CT infections change to 45 RMB. Thus, the estimated lifetime medical cost of CT infections in patients 18-49 years of age in Shandong was 264 million RMB, corresponding to 339 RMB for each female CT infection and 97 RMB for each male CT infection (Table S2). Thus, the estimated range for the total lifetime costs of CT infections in patients 18-49 years of age in Shandong was 172-374 million RMB in 2016. The estimated range of medical costs for each female and male CT infection was 339-361 RMB and 97-108 RMB, respectively.

Table S2 Sensitivity analysis of CT infections cost in patients 18-49 years of age in Shandong

|  | **Cost for each female** | **Cost for each male** | **Total cost** |
| --- | --- | --- | --- |
| CT prevalence | | | |
| Females: 1.5%, Males: 1.6% | 348 RMB | 102 RMB | 172 million RMB |
| Females: 3.2%, Males: 3.8% | 348 RMB | 102 RMB | 374 million RMB |
| Rate of asymptomatic CT infections | | | |
| Ninety percent [21] | 361 RMB | 108 RMB | 285 million RMB |
| Diagnosis methods | | | |
| Antigen detection | 339 RMB | 97 RMB | 264 million RMB |

*Abbreviations*: *CT* Chlamydia trachomatis, *RMB* Renminbi
